# Hybrid System for Engagement Recognition During Cognitive Tasks Using a CFS + KNN Algorithm

**DOI:** 10.3390/s18113691

**Published:** 2018-10-30

**Authors:** Fadilla Zennifa, Sho Ageno, Shota Hatano, Keiji Iramina

**Affiliations:** 1 Graduate School of Systems Life Sciences, Kyushu University, 3-1-1 Maidashi, Higashi-Ku, Fukuoka-Shi, Fukuoka 812-8582, Japan; 3sl17058g@sls.kyushu-u.ac.jp (S.A.); 3sl18055r@s.kyushu-u.ac.jp (S.H.); iramina@inf.kyushu-u.ac.jp (K.I.); 2 Faculty of Information Science and Electrical Engineering, Kyushu University, 744 Motooka, Nishi-Ku, Fukuoka-Shi, Fukuoka 819-0395, Japan

**Keywords:** electroencephalography, electrocardiography, electrooculography, eye tracking, near-infrared spectroscopy, engagement recognition, KNN, CFS, hybrid system, sensor

## Abstract

Engagement is described as a state in which an individual involved in an activity can ignore other influences. The engagement level is important to obtaining good performance especially under study conditions. Numerous methods using electroencephalograph (EEG), electrocardiograph (ECG), and near-infrared spectroscopy (NIRS) for the recognition of engagement have been proposed. However, the results were either unsatisfactory or required many channels. In this study, we introduce the implementation of a low-density hybrid system for engagement recognition. We used a two-electrode wireless EEG, a wireless ECG, and two wireless channels NIRS to measure engagement recognition during cognitive tasks. We used electrooculograms (EOG) and eye tracking to record eye movements for data labeling. We calculated the recognition accuracy using the combination of correlation-based feature selection and k-nearest neighbor algorithm. Following that, we did a comparative study against a stand-alone system. The results show that the hybrid system had an acceptable accuracy for practical use (71.65 ± 0.16%). In comparison, the accuracy of a pure EEG system was (65.73 ± 0.17%), pure ECG (67.44 ± 0.19%), and pure NIRS (66.83 ± 0.17%). Overall, our results demonstrate that the proposed method can be used to improve performance in engagement recognition.

## 1. Introduction

Engagement is defined as a cognitive process involving decision making, information gathering, visual scanning, and selectively sustaining attention on a specific event while ignoring other external influences [1]. Knowing a person’s level of engagement is important in order to gain good performance on a specific task [2]. Billeci et al. [3] adapted a wearable integrated electroencephalograph (EEG) and electrocardiograph (ECG) for measuring the change of neurophysiological and autonomic activity between engagement and disengagement states, in subjects exhibiting autism spectrum disorder. By extracting quantitative EEG (QEEG) features from an EEG signal, as well as heart rate and heart rate variability (HRV) from an ECG, they found evidence of differing activity in the engagement and disengagement states, in both the EEG and ECG. Bierre et al. [4] applied near-infrared spectroscopy (NIRS) to assess anterior frontal hemodynamic responses to engagement during three cognitive tasks. In their study, they presented evidence of age-related anterior frontal hemodynamic changes with cognitive demands.

The classification of engagement based on self-reporting and behavior-based information tends to be delayed, sporadic, and intrusive. Performance-based information can be misleading since multiple degrees of tasks could be grouped together with the same level of performance [5]. Conversely, physiological measures do not require overt behavior, can be arranged to have little or no interference with task execution, and can supply information continuously without significant delay. Hussain et al. [6] investigated the activity of physiological signals and facial responses to cognitive load under an emotional stimulus and collected participant ratings from a self-assessment manikin to find the normative ratings in the collection. They investigated the correlation between physiological data and the level of stimulation. They also subsequently compared the accuracy of cognitive load detection with face video features, physiological features, and participant rating features with fusion features. They concluded that classification with fusion features (i.e., not only based on self-report) performed with more accuracy. Furthermore, measuring human mental states based on physiological activity has also been investigated by Stikic et al. [1]. By integrating EEG and ECG features, they applied an unsupervised method for cognitive state recognition. However, the unsupervised learning requires large amounts of data to get an appropriate pattern. In our study, data labeling relied on physiological activities. We used electrooculograms (EOG) and eye tracking to record eye movements, such as blink rate and pupillometry of participants. Several studies demonstrate that blink rate and pupillometry are correlated with engagement components [7,8,9,10,11,12,13]. The information from the EOG and eye tracker will be used for labeling data in the model data. We classified high engagement level and low engagement level.

We are currently implementing a hybrid technology system that can be used to measure engagement recognition levels during cognitive tasks. Several studies on hybrid system have mentioned their promising characteristics. Ahn et al. [14] have suggested computational integration methods. In their review, they mentioned the multimodal systems of EEG and NIRS, which are high-density types. Hong et al. [15] focused on the utility of the integration between EEG and NIRS for locked-in syndrome patients. They mentioned that the proper selection of features will improve the accuracy of classification. In our study, we investigate the best features and the most common features that can be used in engagement recognition; the difference lies in the approach of the study. Ahn et al. [16] combined EEG, ECG, and NIRS by using 68 electrodes for EEG, ECG, and EOG and 8 channels in the NIRS in simulated driving. In our study, we use a two-electrode EEG, an ECG, and two channels in the NIRS. All mentioned sensors are wireless sensors. Our previous work [17,18] used this system for monitoring the cognitive state in children with developmental disorders during a 7 year training period. This time we would like to do engagement recognition of the low-density hybrid system.

We investigate nine types of linear and nonlinear features from EEG, ECG, and NIRS to find the most common features that can be used in engagement recognition. The investigation of linear and nonlinear features has been previously studied for mental state recognition but in stand-alone systems, such as only for EEG or ECG [19,20,21]. In our study, we tried to adapt these features in the hybrid system. This step was improved by combining the feature selector and classifier. We used the correlation-based feature selection (CFS) introduced by Hall [22] as the feature selector and k-nearest neighbor (KNN) as the classifier, following several comparisons with other classifiers. Although a CFS and KNN combination (CFS + KNN) algorithm with two types searching method (i.e., best first search and greedy stepwise search) has been used by Hu et al. [23], our study applied a CFS + KNN algorithm in a low-density hybrid system and only used one searching algorithm. We decided to use only one search method (i.e., best first search) after demonstrating that this searching method had the highest performance. To our knowledge, this is the first study to employ a hybrid system (EEG, ECG, and NIRS) with fewer than 10 channels and to apply a KNN classifier with a CFS feature selector for engagement recognition.

## 2. Systems Design Section

### 2.1. Participants

There were 18 participants in our experiment. All participants were Kyushu University students, with ages ranging from 21 to 28 (24.3 ± 2.3). All participants had normal visual function and were free of disability; 16 were right handed, and two participants were left handed. Participants were instructed not to consume any caffeine 2 h before the experiment because it could affect the HRV [24,25]. The study was conducted in accordance with the ethical principles of Kyushu University and the Declaration of Helsinki. Written informed consent was obtained from each participant before the experiment.

### 2.2. Engagement Tasks

The experiment was done between 10:30 a.m. and 1:30 p.m. Testing took place in a dimly lit room. We also recorded the behavior activities using a webcam camera (Logicool C270, Logitech, Lausanne, Switzerland), which was located in front of the participant’s face.

Three types of engagement task were used: backward digit span (BDS) [2], forward digit span (FDS) [2], and arithmetic. These tasks are consisted of three level. Level one consisted of series 30 sets of four digits, level two: 30 sets of five digits and level three: six digits. Most of the questions in this experiment were relatively simple and did not require any prerequisite knowledge or specific skills. However, a good level of attention and alertness was required to avoid making easy mistakes because the response time was limited to 20 s. Each trial started with the presentation of a central, white fixation dot on a dark background until the participant’s eyes could be accepted by the eye tracker. Next, cognitive questions (i.e., encoding session) would appear for 10 s and the participant was instructed to respond within 20 s. All cognitive tasks were counterbalanced. The measurement was recorded after the practice session finished.

#### 2.2.1. BDS (Backward Digit Span)

**Figure 1:**
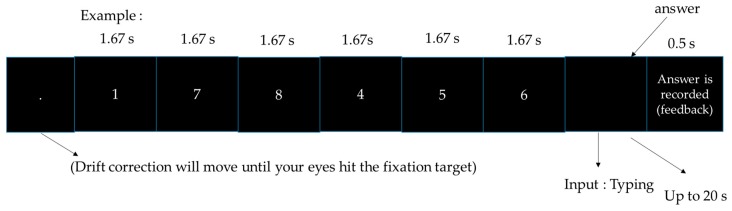
Backward digit span.

In this task, digits would appear within 10 s and participants were asked to type the digits backward in reverse order. The task can be seen in Figure 1.

#### 2.2.2. FDS (Forward Digit Span)

**Figure 2:**
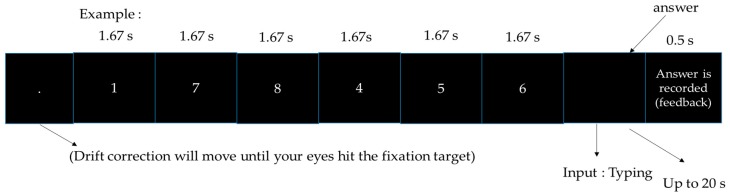
Forward digit span.

In forward digit span, digits would appear within 10 s and the participant is asked to type the digits in the forward order. The task can be seen in Figure 2.

#### 2.2.3. Arithmetic

**Figure 3:**
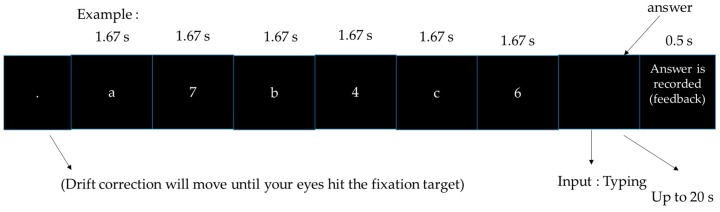
Arithmetic.

For the arithmetic task, a number would appear within 10 s together with a letter. The participants were asked to calculate operations using just the number. The question would appear together with the blank forms, and the participants were asked to type the answer within 20 s. The task can be seen in Figure 3.

### 2.3. Software and Apparatus

Stimuli were presented on a 17-inch CRT monitor (1024 × 768). Testing took place in a dimly lit room. Stimuli presentation was done using OpenSesame [26], using the legacy back-end for the display control and the PyGaze toolbox [27] for the eye tracker.

#### 2.3.1. Eye Tracking

Before the start of each task, participants were positioned in front of an eye tracker (The EyeTribe tracker version 1, Copenhagen, Denmark). The distance of the participants’ eyes from The EyeTribe was estimated to be ~57 cm. The participants were asked to fix their heads on a chin rest. Eight participants were successfully calibrated in the 60 Hz mode, and three participants were successfully calibrated in the 30 Hz mode. In this study, we calibrated and validated the eye tracking system to each participant using a nine-point dot matrix. After validation, the eye tracker that had been embedded with the OpenSesame software labeled each calibration point with the error in the degree of the visual angle between the calibrated and validated measures. If the calibration points do not exceed 1° and the greatest single point error does not exceed 1°, the process would continue. Before each trial, a one-point eye tracker recalibration was performed.

#### 2.3.2. Electrophysiology

**Figure 4:**
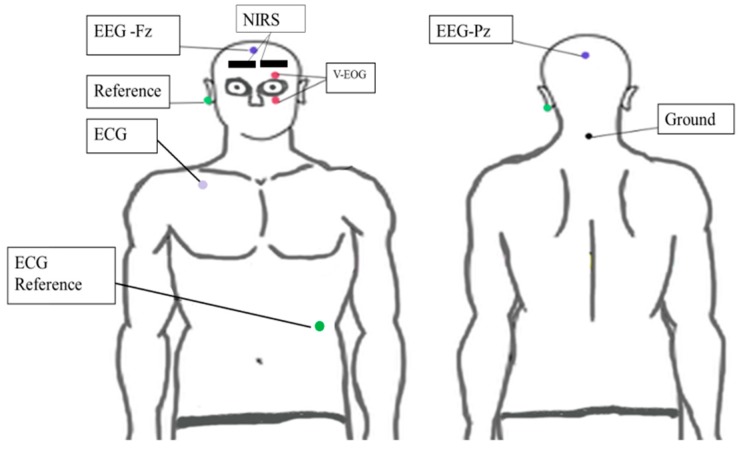
Hybrid system placement: EEG (Fz and Pz), ECG (2-lead placement), NIRS (Fp1 and Fp2).

In this study, EEG, EOG, and ECG (Polymate Mini AP 108, Miyuki Giken Co., Ltd., Kasugai-city, Japan) signals were sent by Bluetooth to a computer. The frequency of sampling was 500 Hz. To evaluate engagement recognition during a cognitive task, we recorded EEG at the Fz and Pz, referenced at A1. These areas are highly correlated in cognitive activities [28,29,30]. The ECG was recorded on the chest (2-lead placement) [1,17,18]. We chose this position for the ECG to reduce the effect of artifact movements when the participant responded to the tasks. We also put two electrodes for a vertical EOG. Figure 4 shows the electrode placements.

#### 2.3.3. Near-Infrared Spectroscopy

A spatially resolved continuous wave NIRS system (PocketNIRS; DynaSense Inc., Hamamasu, Japan) was placed symmetrically to measure hemodynamic activity from the prefrontal region (Fp1 and Fp2). A black tensor bandage was wrapped around the subject’s head to prevent light from entering the sensors. The NIRS signal was sent via Bluetooth to the computer. This NIRS had wavelengths of 735, 810, and 850 nm. The frequency sampling was 10.2 Hz. The NIRS position is shown in Figure 4.

## 3. Analysis for Engagement Recognition

During the answer session, participants would shift their gaze to the keyboard. This condition would also cause artifact movement. So, to ensure high-quality data, we only analyzed the encoding session. The details of our analysis design are as follows.

### 3.1. Data Preprocessing

#### 3.1.1. Pupillometry

Pupillometry is concerned with changes in pupil size. The diameter of the pupil size has long been known as a marker of cognitive load and attentional performance. A study by Van Den et al. [7] mentioned that pupil size could be used to track the focus of attention. In this study, we analyzed pupillometry using a handmade program written in Matlab 2017b. We applied a bandpass filter to avoid from high frequency and calculated the mean value and Z score, as shown in Equation (1):

Z_pupillometry_ = (μ_baseline_ − μ_ptask_)/sd_ptask_ Z_pupillometry_ = Z score of pupillometry μ_baseline_ = mean baseline μ_ptask_ = mean of pupil size during the encoding time sd_ptask_ = standard deviation of pupil size during executing the tasks (1)

#### 3.1.2. Blinking Rates

Blinking has been correlated with cognitive activity [15]. In this study, eye blinks were detected with vertical EOG. To analyze the EOG signal, we used MATLAB 2017b. We performed baseline drift removal. The EOG signal is characterized by a frequency range of 0.1 to 20 Hz, and the amplitude lies between 25 and 3500 μV. We applied a bandpass filter from 0.1 to 20 Hz. We selected the detected peak at more than 50 μV as the criterion [12] for eye blinking. After that, we calculated the Z score on the data. Equation (2) shows how to calculate the *Z* score:

Z_blinkrate_ = (μ_baseline_ − μ_blinkratetask_)/sd_blinkrate_ Z_blinkrate_ = Z score of blink rate μ_blinkratetask_ = mean of blink rate during encoding time μ_baseline_ = mean baseline sd_blinkrate_ = standard deviation of blink eyes during executing the tasks (2)

#### 3.1.3. Engagement Recognition

After calibrating participant eye movements, a 90 s baseline was recorded for each participant [13]. During this time, the participants were asked to relax; it was a neutral situation. After the data were collected, we performed offline analysis. In our study, data labeling is classified into two classes: high engagement and low engagement. We used supervised learning to do the data mining.

##### Data Labeling for Training Data

In our study, we adapted a supervised learning method, which made data labeling a crucial part for definition. Data labeling in this study was based on eye blinks and pupil size. Blinking rates were recorded from the EOG, and pupil size was recorded by the eye tracker. We used the Z score from Formulas (1) and (2). Afterwards, we assigned a point to every datum based on the criteria in Table 1.

Following the scoring, we divided the data into high engagement and low engagement. If the total score between the pupil and blinking indices were greater than 0, we classified the datum as high engagement, and if it was less than 0, we classified it as low engagement.

### 3.2. Feature Extraction

In our features, we combined the results from the nonlinear and linear analysis. In total, there were 59 features in this study (i.e., 34 features from EEG, 7 features from ECG, and 18 features from NIRS). The feature extraction was calculated using a handmade program written in MatLab 2017b.

Table 2 shows the types of features that we used in this study. From the linear parameters, we used the Hjorth parameter to investigate the signals (y(t)) (EEG, ECG, NIRS), based on their activity (Equation (3)), mobility (Equation (4)), and complexity (Equation (5)). The Hjorth parameter has been used in several EEG studies [19,31,32]. Mostly it was used because this parameter is of minimal complexity and calculated in real time. In our study, we used it for the EEG, ECG, and NIRS:

activity = var(y(t))(3)

(4) $$\mathrm{mobility}\;=\sqrt{\left(\left(\mathrm{var}\left(\left(d_{y}\left(t\right)\right)/\mathrm{dt}\right)\right)/\left(\mathrm{var}\left(y\left(t\right)\right)\right)\right)}$$

complexity = (Mobility((d_y_ (t))/dt)/(mobility(y(t))) (5)

The Kolmogorov complexity is an effective way to calculate signal complexity [32]. We used this parameter on the EEG and ECG signals. The EEG signal was calculated in 10 s windows. A third-order Butterworth filter with a frequency cutoff of 0.5 to 65 Hz has been used to filter the data. After applying the filter, Wavelet Daubichies-8 was applied in order to get the value of power spectral density. After obtaining the power of the EEG oscillation, the relative power of each band (i.e., theta (θ) (Equation (6)), alpha (α) (Equation (7)), beta (β) (Equation (8)), and gamma (Equation (9)) were computed from each electrode:

Relative θ = (power θ)/(power θ + power α + power β + power γ)(6)

Relative α = (power α)/(power θ + power α + power β + power γ)(7)

Relative β = (power β)/(power θ + power α + power β + power γ)(8)

Relative γ = (power γ)/(power θ + power α + power β + power γ)(9)

We calculated the change of RR interval of the ECG for every 10 s window. Then, we calculated the value of HRV activity by using a fast Fourier transform. In this study, we calculated the high frequency (HF) ECG component to measure the level of parasympathetic nerve activity in the autonomic nervous system. The HF component can be found from 0.15 to 0.4 Hz. Heart rate (HR) was also calculated, using Equation (10). The maximum power spectral density and power density integral were calculated after obtaining the value of the power spectral density:

HR = 60/((median(RR interval))/(frequency sampling))(10)

We calculated the spectral entropy from EEG and ECG data in a nonlinear domain. This allows the system to be quantified using the rate of information loss or generation and calculated the system randomness, regularity, and predictability.

### 3.3. Predictive Modeling

**Figure 5:**
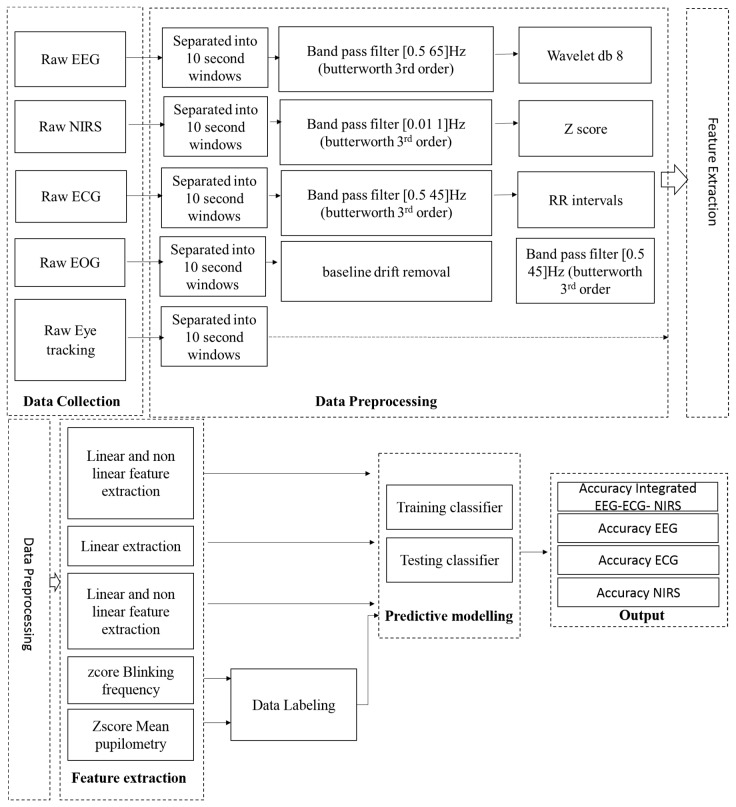
Overview of systems analysis.

We used Weka 3.8 [32] data mining for machine learning. We divided the dataset into two datasets, using 70% from each participant as the training set; testing sets were chosen as a contiguous 30% portion, from each participant’s dataset. In our study, the total sample from each participant was 261 samples, with approximately 182 samples for the training data. The total sample for our training data exceeded 2000 samples. For the first step, we investigated the best validation method by comparing cross-validation methods with several folds; we also investigated the validation methods according to the “leave one participant out” cross-validation (LOSOXV) and hold out validation. Afterwards, we investigated the machine learning algorithm when the system, by using a feature selector and omitting selectors, and investigated when this feature selector is combined with several classifiers such as KNN and support vector machine (SVM). After we found the best performing algorithm, we applied it in the comparison between the hybrid and stand-alone system for engagement recognition. An overview of the analysis system is shown in Figure 5.

The feature selector is important for reducing the time needed to find the best features to be used in a study case [5,22], and it also can increase the accuracy of classification. The CFS is a simple filter algorithm that ranks feature subsets according to a correlation-based heuristic evaluation function [5]. This feature selector calculates features that are highly correlated with the class and uncorrelated with each other. Irrelevant features would be ignored because they have low correlation with the class. Redundant features would be screened out, as they are highly correlated with one or more of the remaining feature. The acceptance of a feature will depend on the extent to which it predicts classes in an area if the instance space was not already predicted by other features. equations (11) through (13) show the calculation process:

M_s_ = (k(r_cf_))/√(k + k(k − 1)(r_ff_)) (11)

CFS = max¦s [(r_(cf1_) + r_(cf2)_ +⋯+ r_(cfk)_)/√(〖k + 2(r_(f1f2)_ + ⋯+ r_(fifj)_ +⋯+ r_(fkf1)_))] (12)

(13) $$\text{CFS}=\begin{array}{c} max\\ x\epsilon \{0,{1\}}^{n} \end{array}\left[\frac{{\left(\sum_{i=1}^{n}a_{i}x_{i}\right)}^{2}}{\sum_{i=1}^{n}x_{i}+\sum_{i\neq j}^{n}2b_{ij}x_{i}x_{j}}\right]$$

M_s_ = Heuristic “merit” of a feature subset, S, containing k features.
r_cf_ = Mean feature-class correlation.
r_ff_ = Average feature-feature intercorrelation.
*k* = Number features.

**Figure 6:**
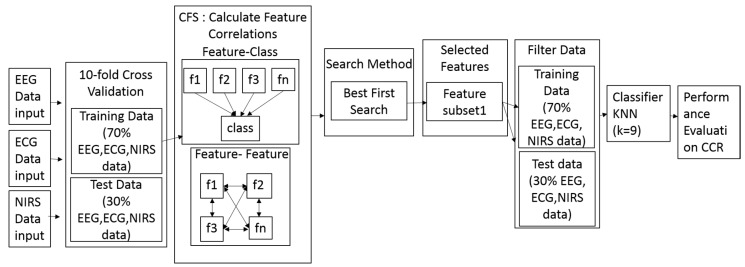
The flowchart of predictive modeling using a CFS and KNN combination (CFS + KNN) method.

This study used a KNN algorithm, which is an approach for data classification that estimates the probability that a data point belongs to one group or another, depending on the group membership of the data points nearest to it. The general details of the predictive learning in our study can be seen in Figure 6.

In this study, the accuracy of performance was calculated on the basis of several variables, such as the true positive (TP) rate. The TP rate was calculated as the proportion of cases that were correctly classified as class high or low among all cases that are truly of the same corresponding class, i.e., the extent to which part of the classes was captured. The TP rate value is also equivalent to the recall. We also calculated the false positive (FP) rate. The FP rate is the proportion of cases that were classified as class high or low but belong to a different class, among all cases that are not of class high or low. The precision is the proportion of the cases that truly have a class low or high among all those that were classified as class high or low. The recall (i.e., sensitivity) is the fraction of relevant instances that have been retrieved over the total amount of relevant instances. The F-measure is simply twice time value of the precision and recall divided by the sum value of precision and recall. We also investigated the area under a receiver operating curve (ROC) for our performance evaluation.

## 4. Results

### 4.1. Classification Algorithm

We adopted several classifiers and investigated the best verification strategy for evaluating recognition performance. We also investigated the best feature selector that decreases the time combination for finding the best features. The feature selection was conducted on the training set, and then the performance was evaluated on the test set. This procedure was iterated until each participant’s data had been tested. This strategy can eliminate the risk of overfitting. Due to technical problems and participant health conditions, 11 participants were analyzed in this study. The sample statistics of engagement level can be seen in Table 3. The sample statistic for the high engagement level is higher than for the low engagement level. The percentage of low engagement level classes is 38% and that of high engagement is 62%.

Because the data were imbalanced, we used balancing filter in Weka 3.28 [33], to balance the sample data. This filter reweighs the instances in the data so that each class has the same total weight. The total sum of weights across all instances will be maintained. Only the weights in the first batch of data received by this filter were changed. The result can be seen in Table 4. The balancing filter increased the weight of sample numbers for the low class and decreased the number of weight in the high class, resulting in the percentage in each class becoming 50%.

To ensure the effect of data balancing, we calculated the accuracy when all 59 features were used and also when only selected features were used and compared the results (Table 5). The use of a balancing filter and the combination of the CFS and KNN have shown the highest accuracy, compared with other methods.

We further examined the usability of the feature selector of the CFS and its search method with other classification algorithms. Table 6 shows the comparison between the CFS and classifiers. In this step, we tried to compare between the combinations of a CFS and a support vector machine (SVM), and CFS + KNN, with several values of k. From the results, we conclude that the combination of CFS and KNN, with a k value of 9, performed the best.

### 4.2. Comparison of Stand-Alone and Hybrid Systems

After determining the best algorithm, we adopted that algorithm in the hybrid system. We calculated the recognition classification from each class. As shown in Table 7, the precision (low = 0.735, high = 0.771) for each class became the highest in the hybrid system, from among the others, during initial training. Other performance calculations in initial training, such as the ROC area, have shown that the hybrid system has the highest value compared with stand-alone systems.

We evaluated the accuracy when the algorithm was applied in stand-alone system by using the CFS + KNN algorithm. As shown in Table 8, the hybrid system achieved the highest accuracy (71.65 ± 0.16%).

We tested our data by comparing the results of the SVM and KNN + CFS classifier, which extended our final choice for engagement recognition with the hybrid system. From our investigation, the standard deviation of the SVM, among participants, (SD = 0.2) is higher than that of the KNN method (SD = 0.16). The details can be seen in Table 9.

## 5. Discussion

In this study, we explored a novel way of combining EEG, ECG, and NIRS with a low-density of electrodes/channels for engagement recognition. The combination of these three different approaches is commonly termed a hybrid system. The integration of NIRS and EEG is complementary, because they enable simultaneous analysis of the neuronal and hemodynamic components of brain activity and do not interfere with each other [34,35]. Hybrid systems, especially low-density hybrid systems, could be effective for engagement recognition under study conditions. They are also practical, especially in a naturalistic condition. In a previous study, we implemented a hybrid system to study intellectual disability child during cognitive training [17,18].

In this study, we sought to implement a low-density hybrid system for engagement recognition during cognitive tasks. To reach our goal, there were several issues we solved, for example, the problem of data imbalance during data labeling. The effect of data imbalance could cause overfitting, and this imbalance was solved by using the class balancer in Weka 3.8 data mining. This method was chosen because other methods, such as under sampling, would prevent the classifier from learning the character of excluded data instances [29]. Afterwards, we also investigated the best validation method to be adopted in this study, in the hope of minimizing the overfitting. We chose the cross-validation method after testing a 10-fold, 3-fold, 5-fold, leave one participant out (LOSOXV), and hold out methods; the result showed that the accuracy in the 10-fold cross-validation method is higher than other classifiers. On the other hand, cross validation method also saving the time of validation. Hu et al. [23] also mentioned cross-validation is the best method for validation, and they used 3-fold cross-validation. In our study, we chose a 10-fold cross-validation rather than a 3-fold cross-validation because it allows the training set to contain 90% of the data instances and the validation set contains the other 10%. With cross-validation, our trained model did not over fit to a specific training subset but rather had the ability to learn from each data fold.

The other issue we tried to solve was the generalizability of features across participants. Li et al. [19] offered a way to find EEG features in cross-participant emotion by exploring 18 kinds of linear and nonlinear EEG features. They examined the effectiveness of these 18 features from a dataset of emotional analysis, using physiological signals and a STJU motion EEG dataset. Their results showed that the considered Hjorth parameter was suitable for analyzing EEG signals. In their evaluation, they found the Hjorth parameter in the beta rhythm led to the best mean recognition accuracy in cross-subject emotion recognition. In our study, we used nine types of linear and nonlinear features to find the most common feature to be used in engagement recognition. As shown in Table 10, the Hjorth parameter becomes the most selected feature by CFS + KNN. Oh et al. [33] applied the Hjorth parameter for extracting EEG features. They found that the Hjorth parameter increased their EEG classification by 4.4%, on average. Following that, we suggest that the Hjorth parameter could be a useful feature for EEG, ECG, and NIRS in engagement recognition. The results from the feature selection also showed that nonlinear parameter features (e.g., spectral entropy) were not chosen as features for engagement recognition either stand-alone system or hybrid system.

Following feature extraction, we calculated a predictive model by using several classifiers. The KNN with k = 9 was selected in this system, after we compared it with other kernels and other classifiers. For example, we investigated SVM (poly kernel), but its accuracy performance was lower (70.78 ± 0.19%) than that of the KNN (71.65 ± 0.16%). The KNN classifier mentioned in the study by Hu et al. [23] had the highest accuracy among other classifiers, especially when combined with a CFS feature selector. In their study, the obtained k value for their systems is 3. In our study, we tried to investigate the KNN classifier with several k values (k = 1, k = 3, k = 9). To be thorough, the KNN with k = 1 has an accuracy of 65.64 ± 0.14%; the k = 3 has an accuracy of 70.34 ± 0.16%; the k = 9 has an accuracy of 71.65 ± 0.16%. The implementation of k = 1 was mostly full of noise. This is because the larger the k value, the more smoothing takes place. From this result, we decided to implement the KNN method with k = 9. Palaniappan et al. [36] compared the performance of SVM and KNN for diagnosing respiratory pathologies. Their result also showed that KNN has a better accuracy than SVM. Although the combination of a CFS and KNN algorithm has been used by Hu et al. [23] for EEG attention recognition, their study used two types of search methods (i.e., best first search and greedy stepwise search). In our study, we applied this algorithm to a low-density hybrid system for engagement recognition during cognitive tasks and only used one searching algorithm. We decided to use only one search method (i.e., best first search) after we found the best first search performed better compared with the greedy stepwise search.

Our algorithm performance was evaluated using the Matthews correlation coefficient (MCC). Based on Chiccho [37], if the MCC value is closer to +1, it means the testing algorithm exhibits a better performance. Conversely, if the value is closer to −1, it means the testing algorithm exhibits a worse performance. From those criteria, the MCC value of our proposed hybrid systems showed the highest values among all other systems. The precision-recall curve (PRC) for our proposed hybrid system also showed the highest value among others. The detail can be seen in Table 11.

**Figure 7:**
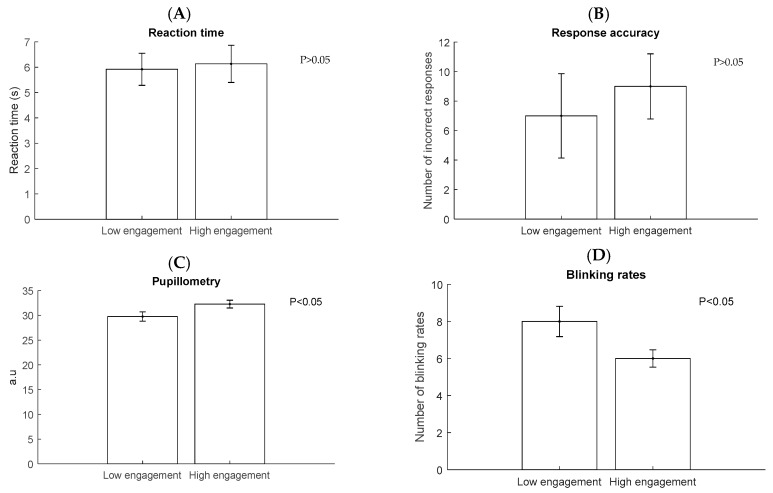
Mean ± SE of reaction time (**A**), incorrect response (**B**), pupilometry (**C**) and Blinking rates (**D**) in high engagement and low engagement (*n* = 11).

Performance of participants for high engagement and low engagement experiments is shown in Figure 7. There were no significant differences in high engagement and low engagement compared with reaction time (*p* > 0.05) and response accuracy (*p* > 0.05) from 11 participants, based on t-test. In the other hand, blinking rates (*p* < 0.05) and pupillometry (*p* < 0.05) shown significant differences in both states. This could be happened because our task contain multiple levels. Level one consisted of series 30 sets of four digits, level two: 30 sets of five digits and level three: six digits. Aghajani et al. [5] mentioned performance-based information as data labeling can be misleading since multiple level of tasks could be grouped together with the same level of performance. This could explain the reliability of physiological data in our recognition.

However, there are still some limitations in this study. We have not studied the effect of fatigue to the brain activities. In future studies, we will also investigate the fatigue effect. Due to the small participant pool, sampling could be a limitation of this study. Even though the small number of participants used for training (*n* = 11) may limit our conclusions, the preliminary results demonstrated the capability of a low-density hybrid EEG, ECG, and NIRS system for use in engagement recognition.

## 6. Conclusions

This study sought to investigate the usability of a low-density hybrid system in engagement recognition. In our investigation, we used a 10-fold cross-validation to validate this hybrid system, after comparing it with other validation methods. We considered using CFS + KNN for engagement recognition. CFS + KNN showed the highest accuracy compared with other selector combinations; the value was 71.65 ± 0.16%. When comparing the result between hybrid and stand-alone system, the hybrid system showed the highest accuracy. We also found that the Hjorth parameter was useful for engagement recognition. From this, we concluded that hybrid system can be validated for engagement recognition.

## Figures and Tables

**Table 1:** Scoring criteria engagement index based on Z score.

Point	Pupil	Blinking
+1	Z score ≤ 0	Z score ≥ 0
−1	Z score > 0	Z score < 0

**Table 2:** Feature types.

Feature Type	Extracted Features
Time-frequency domain features	Hjorth parameter: Activity (EEG, ECG, NIRS) Hjorth parameter: Mobility (EEG, ECG, NIRS) Hjorth parameter: Complexity (EEG, ECG, NIRS) Kolmogorov complexity (EEG, ECG) Maximum power spectral alpha (EEG) Maximum power spectral theta (EEG) Maximum power spectral beta (EEG) Maximum power spectral gamma (EEG) Power density integral alpha (EEG) Power density integral theta (EEG) Power density integral beta (EEG) Power density integral gamma (EEG) Relative power alpha (EEG) Relative power theta (EEG) Relative power beta (EEG) Relative power gamma (EEG) Heartrate (ECG) HF (High frequency value from heart rate variability (ECG))
Nonlinear domain feature	Spectral entropy (EEG, ECG)

**Table 3:** Sample statistic of engagement level.

Engagement Level	Sample Numbers	Weight	Percentage
Low	763	763	38%
High	1250	1250	62%

**Table 4:** Sample statistics of engagement level after using balancing filter.

Engagement Level	Sample Numbers	Weight	Percentage
Low	763	1006	50%
High	1250	1006	50%

**Table 5:** Feature selection method on engagement recognition.

	Balancing + CFS + KNN	KNN	Balancing + KNN
Accuracy	71.65 ± 0.16%	71.17 ± 0.16%	70.84 ± 0.17%

**Table 6:** Feature selector and classifier.

	CFS + SVM	CFS + KNN (k = 1)	CFS + KNN (k = 3)	CFS + KNN (k = 9)
Accuracy	70.78 ± 0.19%	65.64 ± 0.14%	70.34 ± 0.16%	71.65 ± 0.16%

**Table 7:** Detail accuracy recognition during initial training.

	Class	TP Rate	FP Rate	Precision	Recall	F-Measure	ROC Area
hybrid	Low	0.788	0.284	0.735	0.788	0.76	0.811
	High	0.716	0.212	0.771	0.716	0.743	0.811
EEG	Low	0.748	0.331	0.693	0.748	0.72	0.763
	High	0.669	0.252	0.727	0.669	0.697	0.761
ECG	Low	0.775	0.282	0.733	0.775	0.753	0.809
	High	0.718	0.225	0.761	0.718	0.739	0.81
NIRS	Low	0.701	0.302	0.699	0.701	0.7	0.764
	High	0.698	0.299	0.7	0.698	0.699	0.764

**Table 8:** Accuracy comparison of stand-alone and hybrid.

	Hybrid	EEG	ECG	NIRS
Accuracy	71.65 ± 0.16%	65.73 ± 0.17%	67.44 ± 0.19%	66.83 ± 0.17%

**Table 9:** Accuracy performance of each system to each participant.

Participant	SVM	KNN
S1	74.36%	75.64%
S2	88.46%	85.90%
S3	61.54%	61.54%
S4	34.62%	64.10%
S5	51.28%	46.15%
S6	96.15%	96.15%
S7	83.33%	83.33%
S8	78.21%	67.95%
S9	44.87%	50%
S10	70.51%	62.82%
S11	92.31%	91.03%

**Table 10:** Features selected on the basis of CFS attribute evaluator.

	Features Selected
Hybrid	activity (Hjorth Parameter) (fz), power density integral gamma (fz), relative alpha (fz), relative beta (fz), activity (pz), power density integral beta (pz), relative power gamma (pz), complexity ECG (Hjorth parameter), mobility ECG (Hjorth parameter), activity ECG (Hjorth parameter), mobility total (fp1), activity total (fp1), complexity deoxy (fp1), mobility deoxy (fp1), activity deoxy (fp1), mobility tot (fp2), complexity deoxy (fp2), mobility deoxy (fp2)
EEG	Activity (fz), alpha density (fz), theta density (fz), gamma density (fz), Kolmogorov complexity (fz), relative power, relative alpha (fz), relative beta (fz), relative gamma fz, activity (pz), alpha density(pz), beta density(pz), gamma density (pz), relative alpha (pz), relative beta (pz), relative gamma (pz)
ECG	Activity, mobility complexity (Hjorth parameter)
NIRS	Activity [tot(fp1), deoxy(fp1), deoxy(fp2)], mobility [tot(fp1)), deoxy(fp1), tot(fp2), deoxy (fp2)], complexity [tot (fp1), deoxy(fp1), deoxy (fp2) (Hjorth parameter)]

**Table 11:** Evaluation performance.

System	Class	MCC	PRC
Hybrid	Low	0.505	0.781
	High	0.505	0.798
EEG	Low	0.418	0.716
	High	0.418	0.758
ECG	Low	0.493	0.782
	High	0.493	0.795
NIRS	Low	0.399	0.731
	High	0.399	0.751

## References

[1] Stikic M., Berka C., Levendowski D.J., Rubio R.F., Tan V., Korszen S., Barba D., Wurzer D. (2014). Modeling temporal sequences of cognitive state changes based on a combination of EEG-engagement, EEG-workload, and heart rate metrics. Front. Neurosci..

[2] Berka C., Levendowski D.J., Lumicao M.N., Yau A., Davis G., Zivkovic V.T., Olmstead R.E., Tremoulet P.D., Craven P.L. (2007). EEG correlates of task engagement and mental workload in vigilance, learning, and memory tasks. Aviat. Space Environ. Med..

[3] Billeci L., Tonacci A., Tartarisco G., Narzisi A., Di Palma S., Corda D., Baldus G., Cruciani F., Anzalone S.M., Calderoni S. (2016). An Integrated Approach for the Monitoring of Brain and Autonomic Response of Children with Autism Spectrum Disorders during Treatment by Wearable Technologies. Front. Neurosci..

[4] Bierre K.L., Lucas S.J., Guiney H., Cotter J.D., Machado L. (2017). Cognitive Difficulty Intensifies Age-related Changes in Anterior Frontal Hemodynamics: Novel Evidence from Near-infrared Spectroscopy. J. Gerontol. A Biol. Sci. Med. Sci..

[5] Aghajani H., Garbey M., Omurtag A. (2017). Measuring Mental Workload with EEG+fNIRS. Front. Hum. Neurosci..

[6] Hussain M.S., Calvo R.A., Chen F. (2013). Automatic cognitive load detection from face, physiology, task performance and fusion during affective interference. Interact. Comput..

[7] Brink R.L., Murphy P.R., Nieuwenhuis S. (2016). Pupil diameter tracks lapses of attention. PLoS ONE.

[8] Yoder K.J., Belmonte M.K. (2010). Combining computer game-based behavioural experiments with high-density EEG and infrared gaze tracking. J. Vis. Exp..

[9] Mathôt S., Melmi J.-B., Castet E. (2015). Intrasaccadic perception triggers pupillary constriction. PeerJ.

[10] Mathôt S., van der Linden L., Grainger J., Vitu F. (2013). The Pupillary Light Response Reveals the Focus of Covert Visual Attention. PLoS ONE.

[11] Orchard L.N., Stern J.A. (1991). Blinks as an index of cognitive activity during reading. Integr. Physiol. Behav. Sci..

[12] Bulling A., Ward J.A., Gellersen H., Troster G. (2011). Eye Movement Analysis for Activity Recognition Using Electrooculography. IEEE Trans. Pattern Anal. Mach. Intell..

[13] Niehorster D.C., Cornelissen T.H.W., Holmqvist K., Hooge I.T.C., Hessels R.S. (2018). What to expect from your remote eye-tracker when participants are unrestrained. Behav. Res. Methods.

[14] Ahn S., Jun S.C. (2017). Multi-Modal Integration of EEG-fNIRS for Brain-Computer Interfaces–Current Limitations and Future Directions. Front. Hum. Neurosci..

[15] Hong K.-S., Khan M.J., Hong M.J. (2018). Feature Extraction and Classification Methods for Hybrid fNIRS-EEG Brain-Computer Interfaces. Front. Hum. Neurosci..

[16] Ahn S., Nguyen T., Jang H., Kim J.G., Jun S.C. (2016). Exploring Neuro-Physiological Correlates of Drivers’ Mental Fatigue Caused by Sleep Deprivation Using Simultaneous EEG, ECG, and fNIRS Data. Front. Hum. Neurosci..

[17] Zennifa F., Ide J., Noguchi Y., Iramina K. (2015). Monitoring of cognitive state on mental retardation child using EEG, ECG and NIRS in four years study. Eng. Med. Biol. Soc..

[18] Iramina K., Matsuda K., Ide J., Noguchi Y. Monitoring system of neuronal activity and moving activity without restraint using wireless EEG, NIRS and accelerometer. Proceedings of the 2010 IEEE EMBS Conference on Biomedical Engineering and Sciences, IECBES.

[19] Li X., Song D., Zhang P., Zhang Y., Hou Y., Hu B. (2018). Exploring EEG Features in Cross-Participant Emotion Recognition. Front. Neurosci..

[20] Huang S., Li J., Zhang P., Zhang W. (2018). Detection of mental fatigue state with wearable ECG devices. Int. J. Med. Inform..

[21] Zakeri S., Abbasi A., Goshvarpour A. (2017). The Effect of Creative Tasks on Electrocardiogram: Using Linear and Nonlinear Features in Combination with Classification Approaches. Iran J. Psychiatry.

[22] Hall M. (1999). Correlation-Based Feature Selection for Machine Learning. Ph.D. Thesis.

[23] Hu B., Li X., Sun S., Ratcliffe M. (2018). Attention Recognition in EEG-Based Affective Learning Research Using CFS+KNN Algorithm. IEEE/ACM Trans. Comput. Biol; Bioinform..

[24] Martínez-Sellés M., Datino T., Figueiras-Graillet L., Gama J.G., Jones C., Franklin R., Fernández-Avilés F. (2013). Cardiovascular safety of anagrelide in healthy participants: Effects of caffeine and food intake on pharmacokinetics and adverse reactions. Clin. Drug Investig..

[25] de Oliveira R.A.M., Araújo L.F., de Figueiredo R.C., Goulart A.C., Schmidt M.I., Barreto S.M., Ribeiro A.L.P. (2017). Coffee Consumption and Heart Rate Variability: The Brazilian Longitudinal Study of Adult Health (ELSA-Brasil) Cohort Study. Nutrients.

[26] Mathôt S., Schreij D., Theeuwes J. (2012). OpenSesame: An open-source, graphical experiment builder for the social sciences. Behav. Res. Methods.

[27] Dalmaijer E., Mathôt S., Van der Stigchel S. (2014). PyGaze: An open-source, cross-platform toolbox for minimal-effort programming of eyetracking experiments. Behav. Res. Methods.

[28] Culham J.C., Valyear K.F. (2006). Human parietal cortex in action. Curr. Opin. Neurobiol..

[29] Reynolds G.D., Romano A.C. (2016). The Development of Attention Systems and Working Memory in Infancy. Front. Syst. Neurosci..

[30] Chayer C., Freedman M. (2001). Frontal lobe functions. Curr. Neurol. Neurosci. Rep..

[31] Akar S.A., Kara S., Latifoğlu F., Bilgiç V. (2016). Analysis of the Complexity Measures in the EEG of Schizophrenia Patients. Int. J. Neural Syst..

[32] Hall M., Frank E., Holmes G., Pfahringer B., Reutemann P., Witten I.H. (2009). The WEKA data mining software: An update. SIGKDD Explor..

[33] Oh S.H., Lee Y.R., Kim H.N. (2014). A Novel EEG Feature Extraction Method Using Hjorth Parameter. Int. J. Electron. Electr. Eng..

[34] Chen C.-W., Sun C.-W. (2017). Combination of Electroencephalography and Near-Infrared Spectroscopy in Evaluation of Mental Concentration during the Mental Focus Task for Wisconsin Card Sorting Test. Sci. Rep..

[35] Luhmann V.A., Muller K.R. (2017). Why build an integrated EEG-NIRS? About the advantages of hybrid bio-acquisition hardware. IEEE Eng. Med. Biol. Soc..

[36] Palaniappan R., Sundaraj K., Sundaraj S. (2014). A comparative study of the svm and k-nn machine learning algorithms for the diagnosis of respiratory pathologies using pulmonary acoustic signals. BMC Bioinform..

[37] Chicco D. (2017). Ten quick tips for machine learning in computational biology. BioData Min..

